# Effect of inter-pregnancy interval on serum ferritin, haematocrit and pregnancy outcome in Ilorin, Nigeria

**DOI:** 10.4314/ahs.v23i1.35

**Published:** 2023-03

**Authors:** Callistus Elegbua, Hadijat Raji, Sikiru Biliaminu, Grace Ezeoke, Abiodun Adeniran

**Affiliations:** 1 Save a Life Hospital, Lagos, Nigeria; 2 University of Ilorin, College of Health Sciences, Ilorin, Nigeria; 3 Obstetrics & Gynaecology Department, University of Ilorin Teaching Hospital, Ilorin, Nigeria; 4 Department of Chemical Pathology, University of Ilorin Teaching Hospital, Ilorin, Nigeria

**Keywords:** Inter-pregnancy interval, serum ferritin, haematocrit, pregnancy outcome

## Abstract

**Background:**

Available information remains limited on inter-pregnancy interval (IPI) and its effect on maternal health and pregnancy outcome.

**Objectives:**

To determine the effect of IPI on maternal serum ferritin, haematocrit and pregnancy outcome.

**Materials and methods:**

A prospective cohort study of 316 women categorized into WHO recommended IPI of ≥24 months (group I) and IPI <24 months i.e. short IPI (SIPI) as group II after matching for gestational age and social status. Serum ferritin and haematocrit levels were assayed in first and second trimesters; primary outcome measures were maternal serum ferritin, haematocrit and pregnancy outcome gestational age at delivery, birth and placental weights, APGAR scores and neonatal intensive admission). Participants were followed up until six-week post-delivery. Data analysis was with SPSS version 21.0; p<0.05 was significant.

**Results:**

Women in group I had higher mean serum ferritin (37.40±3.15 vs. 32.61±2.68; P<0.001), booking haematocrit (33.24±3.59 vs. 27.92±2.67; P<0.001) and mean birth weight (3100±310 vs. 2700±350; P<0.001). Antenatal hospital admission (P0.002), preterm delivery (P<0.001) and neonatal intensive care admission (P<0.001) were higher for group II. There was no maternal mortality; perinatal mortality was zero (group I) and 95/1000 livebirth (group II).

**Conclusion:**

Low serum ferritin, haematocrit and adverse neonatal outcomes were associated with SIPI.

## Introduction

Pregnancy, childbirth and breastfeeding are stressful events associated with increased physiological, nutritional and psychological demands on the woman. This emphasizes the need for optimal maternal pre-conception health status to attain the required reserves for optimal pregnancy outcome.^1^ However, inadequate pregnancy spacing has been associated with adverse obstetric outcomes including maternal and perinatal morbidity and mortality.^1^–^3^ Inter-pregnancy interval (IPI) is defined as the period between delivery of the previous infant and conception of the current pregnancy; while excluding miscarriage as a preceding pregnancy event.^1^,^2^,^4^

Optimal IPI remained a worldwide obstetric concern and the World Health Organization (WHO) recommended an interval of at least 24 months after a live birth before attempting the next pregnancy.^4^ Short inter-pregnancy interval (SIPI) have been associated with adverse obstetric outcomes with increased risk of preterm births, low birth weight, intrauterine growth restriction and anaemia^1^–^5^ while about one-third of pregnancies in some parts of Africa fall short of the WHO recommended IPI.^6^

he consequences of SIPI have been attributed to maternal depletion syndrome (MDS) which is a biological phenomenon of inadequate maternal recuperation after pregnancy leading to a hostile intrauterine environment for subsequent pregnancy.1 MDS manifests as deficiency of macro and micro nutrients leading to anaemia, osteomalacia, inadequate maternal weight gain in pregnancy, edema, preterm delivery and small for gestational age babies.^1^ Iron and folic acid are central in the regulation of physiological processes taking place during pregnancy. Thus, haematological parameters like the haematocrit which assesses current anaemia and serum ferritin which is a sensitive marker to assess iron stores are strategic in evaluating the relationship between IPI, MDS and adverse pregnancy outcome.^1^ SIPI especially when combined with prolonged breastfeeding results in maternal depletion of micro/macro-nutrients which are not reverted until after 12 months postpartum.^1^ Although maternal serum iron and ferritin fall progressively in pregnancy while free protoporphyrin and transferring receptor levels increase; a markedly reduced serum ferritin (< 12µg/L) remains diagnostic of maternal depletion.^7^ In low-income countries, the combination of poor nutritional status and SIPI in a weak health system raises the probability of the attendant adverse pregnancy outcomes. However, reports on pregnancy outcome relative to IPI are sparse while information on the relationship of IPI to maternal haematological characteristics (serum ferritin and haematocrit levels) is unavailable. Therefore, this study aimed to evaluate the effect of IPI on maternal serum ferritin and haematocrit levels as well as pregnancy outcome among booked parturient women.

## Materials and Methods

### Study setting

The study was conducted at the University of Ilorin Teaching Hospital (UITH) which is a tertiary facility and a major referral centre in North Central Nigeria.

### Study design

A prospective cohort study.

### Study participants

Participants were pregnant women who booked index pregnancy, accessed antenatal services and delivered at the study site.

### Inclusion criteria

The inclusion criteria included live birth from the last pregnancy, antenatal booking of index pregnancy at the study site in the first trimester, singleton pregnancy and no prior use of iron supplement in index pregnancy.

### Exclusion criteria

Women with previous uterine surgery, chronic medical disorders, infective conditions (because inflammation affects serum ferritin values) or those who did not eventually deliver at the study site were excluded from the study.

### Sample size determination

The sample size was determined using the formula for cohort study as shown below^8^



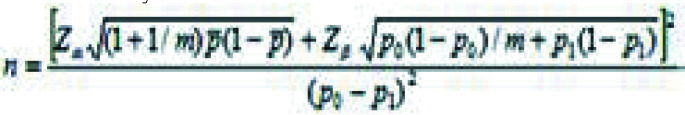





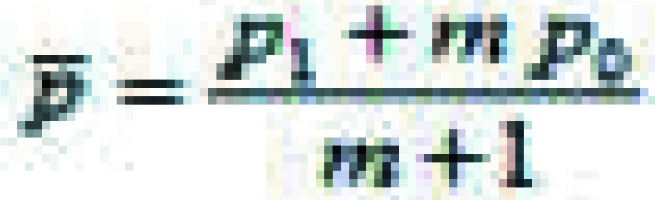



Z α: Standard normal variate for level of significance (usually 5%), = 1.96

Z β: Standard normal variate for power of 1- β. 80% = 0.84

m: The ratio of the study participants on both arm of the study (1:1)

p1: Probability of events in the group below WHO recommended IPI (58.7%).^8^

p0: Probability of events in the group within WHO recommended IPI (79.0%).^8^

p = 58.7 + 1 (79) = 68.85

1 + 1

Thus, N = [1.96 √(1 + 1/1) 68.85(1 – 68.85) + 0.84 √79(1 – 79)/1 + 58.7 (1 – 58.7)]^2^ = 132

(79 – 58.7)^2^

With provision for 20% attrition (26 participants), the sample size for each arm of the study =132+26=158. Thus, the minimum sample size for the study was 316.

### Sampling method

Systematic sampling was employed. First, all pregnant women were screened to determine those who satisfied the inclusion criteria, thereafter, eligible women were evaluated to determine those who satisfied the WHO recommended IPI (group I) and those who had SIPI (group II).

### Study hypothesis

The Null hypothesis for the study states that there is no difference in the serum ferritin and haematocrit levels as well as pregnancy outcome of women within the WHO recommended IPI compared to those with SIPI.

The alternate hypothesis for the study states that that there is a difference in the serum ferritin and haematocrit levels as well as pregnancy outcome of women within the WHO recommended IPI compared to those with SIPI.

### Definition of terms

For the purpose of this study, the following definitions were applied:

- Inter-pregnancy interval (IPI): This referred to the period between the end of the last pregnancy (delivery) and conception of the current pregnancy. 4,5
- WHO recommended inter-pregnancy interval: This referred to inter-pregnancy interval of at least 24months to less than 72months after a live birth.^4^
- Short inter-pregnancy interval (SIPI): This referred to inter-pregnancy interval less than 24months.^4^
- Social class: this was captured using the recommendation of Olusanjo et al which involves summation of the score for the woman's level of education (tertiary=0, secondary=1, primary or less=2) and the partner's occupation (professional=1, semi-skilled=2, unskilled=3). The total represents the social class where 1 is the highest and 5 the lowest.^9^

### Study procedure

#### Recruitment

Recruitment for the study was at the antenatal booking clinic; women who presented for booking were informed about the study by the researchers during the routine group health talk sessions. Those willing to participate were screened for eligibility using the inclusion/ exclusion criteria. Thereafter, a voluntary written informed consent was obtained from each eligible woman after information was provided on the study, the need for blood sample collection, access to the woman and neonate's record (during antenatal period and delivery), the need to deliver at the study site, as well as the use of the data for analysis and publication. The participants were assured of confidentiality and the use of the data obtained solely for research purpose while exit from the study was assured at any time if the woman so desired without any negative consequence on her access to quality care at the facility. Eligible women were thereafter categorized into group I (satisfied the WHO recommended IPI) and group II (IPI less than 24 months).^4^ The gestational age was derived from a first trimester ultrasound scan done for all participants at recruitment into the study by trained sonologists while the IPI was calculated as the time interval from the end of last pregnancy to onset of index pregnancy (date of onset of index pregnancy minus date of delivery of preceding pregnancy) expressed in months. At the study site, booking clinic is conducted once a week; and average of 70 women booked per week while about 24 were in the first trimester. Recruitment was over a six-month period; thus, a sampling interval of one out of every two women who presented in first trimester was used for recruitment with a maximum of 12 recruitments per week.

### Serum ferritin and haematocrit estimation^10^

The standard operating procedure (SOP) for sample collection involved collection of 2ml of venous blood into a plain bottle from the antecubital fossa after application of tourniquet proximal to the site and cleaning the site with alcohol-soaked swab. After sample collection, haemostatsis was secured at the puncture site by pressure application of sterile cotton wool over the site. The sample was allowed to stand for one hour to allow clotting, then centrifuged at 3000rpm for 3minutes. The serum obtained was transferred into another plain bottle, refrigerated at 2 to 8°C for a maximum period of 5days within which it was analyzed. The analysis was carried out using AccuBind^TM^ Ferritin Microplate ELISA using the principle of Immunoenzymometric sequential assay based on antigen-antibody reaction. In this method, ferritin calibrators, patient and control sera were first added to a streptavidin coated well. Biotinylated monoclonal antibody (specific for ferritin) was added and the reactants mixed. Reaction resulted between the biotinylated ferritin antibody and native ferritin to form an immune complex that was deposited on the streptavidin coated well. The excess serum proteins were washed away via a wash step. Another ferritin specific antibody, labeled with an enzyme, was added to the wells. The enzyme labeled antibody bound to the ferritin already immobilized on the well. Excess enzyme was washed off via a wash step. A bluish color was generated by the addition of a substrate which later changes to yellow after adding the stop solution. The intensity of the yellow color generated was directly proportional to the concentration of the ferritin in the sample. Quality control was achieved by having an independent consultant laboratory physician analyze randomly selected 10% of each batch of samples using commercial control sera for validation. All samples were analyzed at the haematology and chemical pathology laboratories of the study site. The blood sample for haematocrit was collected in the EDTA bottles and transferred into capillary tube which was sealed on one end and spun for 3-5minutes in a haematocrit centrifuge; the result was read with micro-haematocrit reader.

### Follow up of participants

All participants had oral iron supplementation from booking till the end of puerperium while postnatal follow up was at the postnatal clinic. All participants were followed up at the antenatal clinic by the researchers and research assistants who were trained resident doctors at the study site. Frequency of antenatal visit was 4-weekly till 28 weeks, 2-weekly till 36weeks and weekly till delivery; monitoring included routine antenatal evaluation as well as serum ferritin and haematocrit at the first and second trimester. Participants were contacted on phone following every missed visit and an alternative visit scheduled. During labour, all participants were monitored with the use of partograph followed by routine standard puerperal care for both mothers and their babies; exit from the study was six weeks postpartum.

### Data management

The data was collected by the researchers and research assistants using the data collection sheet designed for the study. Data collected included maternal socio-demography, obstetric history, antenatal follow up details, onset of labour, mode of delivery, neonatal status at birth, neonatal intensive care admission and management as well as postnatal visit evaluation. Visual validity crosschecks of all data collection sheet were performed by the authors before exit of the participant from the study and missing information obtained. The data was double entered into the computer by the data analyst with an in-built validation procedure which allowed prompt identification and correction of inconsistencies. Data analysis was performed using the Statistical Package for Social Sciences software (SPSS) version 21.0. Results were presented in tables, tests of significance included relative risk for strength of association; t-test was used for mean values, Chi-square for comparison of categorical variables, Fishers' exact test was used as correction for empty cells or cells with low values which did not fulfill the criteria for Chi-square while Mann Whitney U test was used to compare median values of continuous variables.

### Ethical approval

Ethical approval was obtained from the ethical review committee of the University of Ilorin Teaching Hospital (UITH), Ilorin, Nigeria before commencement of the study (Approval number ERCPAN/2016/04/1538). Also, a written informed consent was obtained from all participants at recruitment into the study.

## Results

From figure I, a total of 1,915 women who booked during the study period were screened; 644 were eligible, among these, 316 were recruited as 158 into each arm of the study. In the WHO recommended IPI group (group I), 158 women were recruited; two were lost to follow up while 156 completed the study. In the SIPI group (group II), out of the 158 recruited participants, four were lost to follow up while 154 completed the study. The attrition rate for the study was 1.9% (6 out of 316) and participants who did not complete the study were excluded from the statistical analysis.

**Figure 1:**
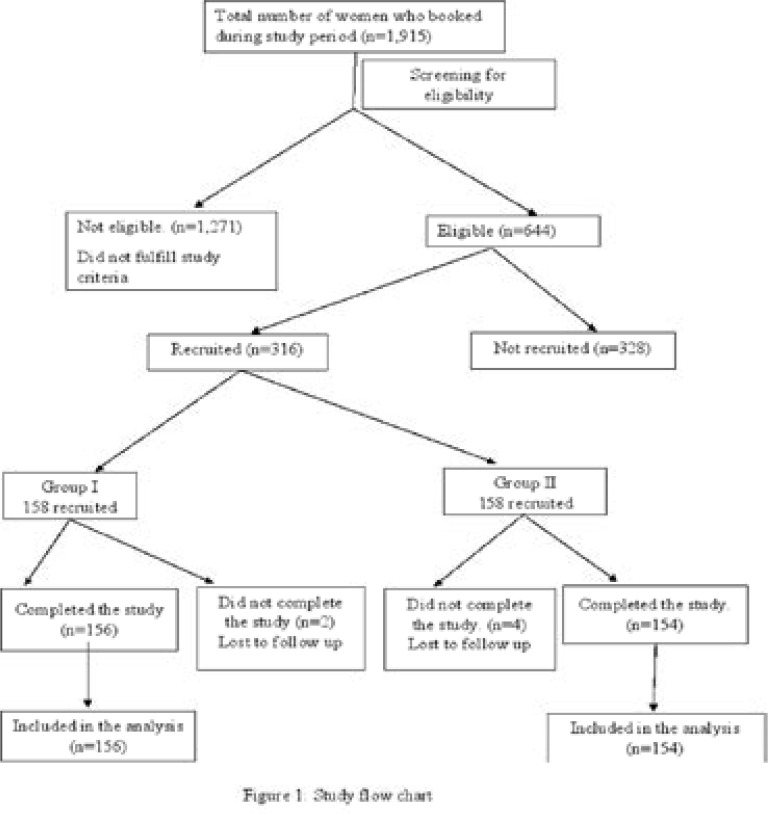
Study flow chart

Table 1 shows that the mean ages were comparable for the two groups (31.62±3.89 vs. 31.35±3.34, P0.449); group I had higher employment status (121 vs. 101; P0.014), tertiary education was commoner (88 vs.37) while secondary (56 vs. 66) and primary (14 vs. 53) education were fewer in group I compared to group II). The gravidity (P0.864), parity (P0.525) and number of living children (P0.825) were not statistically different between the two groups. By study design, the mean gestational age at recruitment (11.45±2.38 vs. 11.31±2.10) and social class were comparable in the two groups.

**Table 1:** Socio-demographic variables of the study participants

Variable	Group I	Group II	χ^2^/t	*p* value
	n=156 (%)	n=154 (%)		
**Age (years)**				
Mean ± SD	31.42 ± 3.89	31.35 ± 3.34		
Range	24 – 42	23 – 40		
**Marital Status**				
Single	3 (1.9)	0 (0.0)	2.991^F^	0.248
Married	153 (98.1)	154 (100.0)		
**Employment**				
Employed	121 (77.6)	101 (65.6)	6.057	0.014
Unemployed	35 (22.4)	53 (34.4)		
**Education**				
Primary	12 (7.7)	49 (31.8)	46.059^F^	0.006
Secondary	56 (35.9)	66 (42.9)		
Tertiary	88 (56.4)	37 (24.0)		
No formal education	0 (0.0)	2 (1.3)		
**Gravidity** 2	51(32.7)	50(32.5)	0.293	0.864
3–4	87(55.7)	89(57.8)		
≥5	18(11.6)	15(9.7)		
**Parity**				
1–2	106(67.9)	113(73.4)	1.142^F^	0.525
3–4	48(30.8)	39(25.3)		
≥5	2(1.3)	2(1.3)		
**Living children**				
1	60(38.5)	60(39.0)	0.384	0.825
2–4	83(53.2)	84(54.5)		
≥5	13(8.3)	10(6.5)		
**Mean GA at booking**	11.45±2.38	11.31±2.10		
**Social class**				
I	48(30.8)	44(28.6)		
II	37(23.7)	35(22.7)		
III	25(16.0)	24(15.6)		
IV	28(18.0)	28(18.2)		
V	18(11.5)	23 (14.9)		

χ^2^: Chi square test; t: Independent samples T test; ^F^: Fisher's exact test; GA: gestational age

Table 2 shows that the mean levels of serum ferritin were higher among group I participants in the first (46.83±3.38ng/ml vs. 35.83±3.05ng/ml; P<0.001) and second trimesters (36.07±3.11ng/ml vs. 32.36±2.66ng/ml; P<0.001) as well as overall mean values (37.40±3.15ng/ml vs. 32.61±2.68ng/ml; P<0.001). Similarly, the mean haematocrit levels were higher for group I participants in the first (32.50±3.02% vs. 28.08±2.91%; P<0.001) and second trimester (33.36±3.67% vs. 27.90±2.66%; P<0.001) as well as the overall values (33.24±3.59% vs. 27.92±2.67%; P<0.001).

**Table 2:** Serum ferritin and haematocrit levels of participating parturient

Variables	Group I	Group II	t	P value
**Serum ferritin (ng/ml)**				
**1^st^ trimester**				
Mean ± SD	46.83 ± 3.38	35.83 ± 3.05	9.373	<0.001
**2^nd^ trimester**				
Mean ± SD	36.07 ± 3.11	32.36 ± 2.66	4.128	<0.001
**Overall**				
Mean ±SD	37.40 ± 3.15	32.61 ± 2.68	-14.558	<0.001
Range	6.00 – 340.00	2.30 – 190.00		
Median (IQR)	43.75 (12.50 – 95.63)	30.00 (20.00 – 60.00)		
**Haematocrit (%)**				
**1^st^ trimester**				
Mean ± SD	32.50 ± 3.02	28.08 ± 2.91	12.515	<0.001
**2^nd^ trimester**				
Mean ±SD	33.36 ± 3.67	27.90 ± 2.66	14.376	<0.001
**Overall**				
Mean ±SD	33.24 ± 3.59	27.92 ± 2.67	14.964	<0.001
Range	28 – 44	23 – 35		
Median (IQR)	32.00 (30.00 – 35.25)	28.00 (25.00 – 30.00)		

t: Independent samples T test

Table 3 shows that medical disorders of pregnancy like pre-eclampsia (2 vs. 4; P0.446), antepartum haemorrhage (2 vs. 5; P0.281), gestational diabetes (1 vs. 5; P0.120), antepartum anaemia (13 vs. 22; P0.107) and antenatal hospital admission (9 vs. 26; P0.002) were higher in group II compared to group I.

**Table 3:** Antenatal follow-up and complications among participants

Variable	Group I	Group II	χ^2^	*P* value
	n=156 (%)	n=154 (%)		
**Pre-eclampsia**				
Yes	2(1.3)	4(2.6)	0.706^F^	0.446
No	154(98.7)	150(97.4)		
**Ante-partum** **haemorrhage**				
Yes	2(1.3)	5(3.3)	1.355^F^	0.281
No	154(98.7)	149(96.7)		
**Gestational diabetes**				
Yes	1(0.6)	5(3.3)	2.772^F^	0.120
No	155(99.4)	149(96.7)		
**Urinary tract infection**				
Yes	5(3.2)	11(7.1)	2.370	0.124
No	151(96.8)	143(92.9)		
**Anaemia**				
Yes	13(8.3)	22(14.3)	2.603	0.107
No	143(91.7)	132(85.7)		
**Antenatal hospital** **admission**				
Yes	9(5.8)	26(16.9)	9.286	0.002
No	147(94.2)	128(83.1)		
**Indication for** **antenatal admission**				
Anaemia	1 (11.1)	9 (34.6)		
Urinary tract infection	4 (44.5)	5 (19.3)		
Hypoglycaemia	0 (0.0)	3 (11.5)		
Severe preeclampsia	2 (22.2)	3 (11.5)		
Haemorrhage	0 (0.0)	4 (15.4)		
Malaria	2 (22.2)	2 (7.7)		

χ^2^: Chi square test; ^F^: Fisher's exact test

From table 4, induction of labour (4 vs. 12; P0.042), preterm delivery (3 vs. 42; P<0.001), first minute APGAR score <7 (15 vs. 50; P<0.001), fifth minute APGAR scores <7 (3 vs.28; P<0.001), need for neonatal resuscitation (4 vs.26; P<0.001), neonatal intensive care admission (2 vs. 28; P<0.001) and mean placen0.014) were higher for group II participants. The mean birth weight (3100±310 vs. 2700±350, P<0.001) was higher in group I participants; neonatal death was zero in group I and 18(11.7%) in group II respectively.

**Table 4:** Pregnancy outcome among participants

Variable	Group I	Group II	χ^2^	*P* value
	n=156 (%)	n=154 (%)		
**Onset of labour**				
Spontaneous	152(97.4)	142(92.2)	4.327^F^	0.042
Induced	4(2.6)	12(7.8)		
**Mode of delivery**				
Spontaneous vaginal	151(96.8)	138(89.6)	6.572^F^	0.015
Instrumental vaginal	0(0.0)	1(0.6)		
Caesarean	5(3.2)	15(9.7)		
**GA at delivery**				
<37	3(1.9)	34(22.1)	29.950^F^	<0.001
37–42	153(98.1)	120(77.9)		
**1st minute APGAR**				
< 7	15 (9.6)	50 (32.5)	24.422	<0.001
≥ 7	141 (90.4)	104 (67.5)		
**5th minute APGAR**				
< 7	3 (1.9)	28 (18.2)	22.762^F^	<0.001
≥ 7	153 (98.1)	126 (81.8)		
**Need for resuscitation**				
Yes	4 (2.6)	26 (16.9)	18.176^F^	<0.001
No	152 (97.4)	128 (83.1)		
**NICU Admission**				
Yes	2 (1.3)	28 (18.2)	25.322^F^	<0.001
No	154 (98.7)	126 (81.8)		
**Final neonatal state**				
Discharged home	156 (100.0)	136 (88.3)	19.358^F^	<0.001
Died	0 (0.0)	18 (11.7)		
**Birth weight (g)**				
Mean ± SD	3100 ±310	2700 ±350	10.815^t^	<0.001
Range	2510 . 4600	1700 . 4600		
Median (IQR)	3000 (2900–3200)	2700 (2400–2.900)	3596.500^U^	<0.001
**Placenta weight**				
Mean ± SD	520 ±40	530 ±50	-2.459^t^	0.014
Range	400 -600	300 -600		
Median (IQR)	500(500–510)	500 (500–600)	10984.000^U^	0.111

χ^2^: Chi square test; ^F^: Fisher's exact test; t: Independent samples T test; U: Mann Whitney U test; GA: Gestational age

Table 5 shows that maternal puerperal complications such as fever (P0.060), pallor (P0.137), foul smelling lochia (P0.121) and anaemia (P0.066) as well as neonatal complications including jaundice (P0.497) and anaemia (P<0.001) were higher among group II compared to group I participants.

**Table 5:** Maternal and neonatal postnatal clinic evaluation outcome among participants

Variable	Group I	Group II	Total	χ^2^	*p* value
	n (%)	n (%)	n (%)	
**Maternal Temperature** **(°C)**					
Mean ± SD	36.9 ± 0.2	36.9 ± 0.3		-1.040^t^	0.299
Range	36.0 -37.4	36.0–38.2			
**Maternal Fever**					
Yes	0 (0.0)	4 (2.6)	4 (1.3)	4.105^F^	0.060
No	156 (100.0)	150 (97.4)	306 (98.7)		
**Maternal Pallor**					
Yes	3 (1.9)	8 (5.2)	11 (3.5)	2.424^F^	0.137
No	153 (98.1)	146 (94.8)	299 (96.5)		
**Abdominal tenderness**					
Yes	0 (0.0)	2 (1.3)	2 (0.6)	2.039^F^	0.246
No	156 (100.0)	152 (98.7)	308 (99.4)		
**Foul smelling lochia**					
Yes	0 (0.0)	3 (1.9)	3 (1.0)	3.069^F^	0.121
No	156 (100.0)	151 (98.1)	307 (99.0)		
**Haematocrit**					
Mean ± SD	31.83 ± 2.31	31.29 ± 2.70		1.894t	0.059
Range	27 – 40	23 – 36			
**Anaemia**					
Yes	1 (0.6)	6 (3.9)	7 (2.3)	3.720^F^	0.066
No	155 (99.4)	148 (96.1)	303 (97.7)		
**Neonatal parameter** **Temperature**					
Mean	36.7±0.2	36.8±0.3		-1.433t	0.153
Range	26.8–37.0	36.0–37.0			
**Jaundice**					
Yes No	0(0.0) 156(100.0)	1(0.7) 153(99.3)		1.016^F^	0.497
**Haematocrit**					
Mean	39.5±2.4	32.6±3.2		20.816t	<0.001
Range	31.0–45.0	27.0–40.0			
**Anaemia**					
Yes	0(0.0)	21(15.4)		22.819^F^	<0.001
No	156(100)	133(84.6)			

χ^2^: Chi square test; ^F^: Fisher's exact test; t: Independent samples T test

## Discussion

This study showed that parturient who satisfied the WHO recommended IPI of 24months or more (group I) had significantly better employment and education attainment, as well as improved maternal health evidenced by higher serum ferritin and haematocrit levels at both first and second trimesters. In addition, women with WHO recommended IPI spaced pregnancies recorded better maternal, fetal and neonatal outcome with no neonatal mortality compared to women with SIPI (group II). The study's strength is the serial evaluation of maternal haematological parameters as well as neonatal and maternal puerperal status. It was limited by the restricted geographical distribution and the sample size.

Higher level of education as observed among participants with WHO recommended IPI in this study has been reported to be associated with good health seeking habits, improved access to health services and a general appreciation of health services.^6^ Conversely, low level of formal education has been associated with SIPI, late booking for antenatal care and poor pregnancy outcome.^6^ Although by its design this study did not assess for late booking, women with SIPI had poor pregnancy outcome compared to appropriately spaced pregnancies.

The observed lower serum haematocrit among pregnant women with SIPI is similar to a previous research report.^6^ The serum ferritin level is a reflection of the iron reserve and is directly related to the haematocrit; thus, the observed trend with lower values among women with SIPI in this study is not unexpected. The lower values may be explained by the concept of maternal depletion syndrome attributed to inadequate recuperation from the physiological and nutritional strain of the preceding pregnancy among women with SIPI.^1^ However, despite the physiologic progressive decrease of serum ferritin levels in pregnancy, women with WHO recommended IPI recorded higher values than those with SIPI during pregnancy. In this study, there was no significant difference in anaemia on follow up evaluation among the participants despite the initial significant difference earlier in pregnancy. This supports the report that supplementation with food and micronutrients for population at risk of maternal depletion syndrome during pregnancy limits adverse pregnancy outcome.^1^

This study shows better neonatal outcome for babies delivered after WHO recommended IPI compared to those from SIPI. Preterm delivery was higher among participants with SIPI in this study similar to other reports.^1^,^2^,^4^,^11^ This has been associated with the insufficient maternal iron level which may translate to foetal low iron / hemoglobin and chronic hypoxia from oxidative stress leading to a hostile intrauterine environment and possible preterm birth.^1^ It was observed that neonates of women with SIPI had perinatal asphyxia evidenced by low APGAR scores, higher need for neonatal resuscitation and neonatal intensive care admissions. This compares to the report of a population study from the USA which reported that SIPI is a significant contributor to neonatal morbidities including risk for assisted ventilation at delivery, low fifth-minute APGAR score and neonatal intensive care admission.^2^ This may be connected to a report that low iron levels leads to increased production of norepinephrine and eventually increased corticotrophin-releasing hormone which limits fetal growth, development and neonatal performance.^1^ Also, all neonatal mortalities were among babies of women with SIPI. Researchers have documented high perinatal morbidities and mortalities among women with SIPI which may be traceable to perinatal asphyxia which is worsened by prematurity among the babies delivered after SIPI.^1^,^2^,^5^ In addition, babies born after SIPI had lower mean birth weight and higher mean placenta weight compared to born after the WHO recommended IPI. While the low birth weight may be explained by prematurity, the increased mean placenta weight could be a compensatory mechanism for fetal intrauterine hypoxia causing placenta hyperplasia to increase uteroplacental circulation.^1^

Maternal puerperal evaluation at the postnatal clinic was comparable after short and appropriately-spaced pregnancies relative to pallor, uterine tenderness, the state of the lochia, haematocrit and anaemia. This could be attributed to the compensatory effect of antenatal and postpartum iron supplementation as well as other puerperal care instituted following hospital supervised care. However, neonatal evaluation at the postnatal clinic showed lower haematocrit in babies born after SIPI. This can be related to the preterm deliveries and the effects of other intrauterine insult suffered by the baby's consequent to the SIPI as an independent risk factor for adverse perinatal and neonatal outcomes.^1^,^12^

However, attributing the adverse pregnancy outcomes following SIPI to maternal depletion theory has been challenged. A population study conducted in the USA, reported that implicating maternal depletion syndrome especially folic acid deficiency in preterm birth from IPI in developed countries like USA where malnutrition is uncommon and fortification as well as supplementation with folic acid is the norm is questionable. The authors opined that instead of maternal depletion as a sole factor, a multi-factorial theory where other factors are evaluated as possible causative agents should be considered especially in developed countries.^11^

This study concludes that SIPI results in adverse maternal and neonatal outcomes compared to pregnancies spaced using the WHO recommendation. Although iron supplementation in pregnancy improved the serum ferritin and haematocrit levels, it did not eradicate the negative effects of the SIPI. The study recommends greater efforts to encourage compliance with the recommend on appropriate spacing of pregnancies to prevent the poor pregnancy outcomes.
